# Catalytic Ozonation of Recalcitrant Organic Chemicals in Water Using Vanadium Oxides Loaded ZSM-5 Zeolites

**DOI:** 10.3389/fchem.2019.00384

**Published:** 2019-05-31

**Authors:** Yingying Xu, Qinghong Wang, Brandon A. Yoza, Qing X. Li, Yue Kou, Yuqi Tang, Huangfan Ye, Yiming Li, Chunmao Chen

**Affiliations:** ^1^State Key Laboratory of Heavy Oil Processing, State Key Laboratory of Petroleum Pollution Control, China University of Petroleum, Beijing, China; ^2^Hawaii Natural Energy Institute, University of Hawaii at Manoa, Honolulu, HI, United States; ^3^Department of Molecular Biosciences and Bioengineering, University of Hawaii at Manoa, Honolulu, HI, United States

**Keywords:** ZSM-5 zeolites, catalytic ozonation, vanadium loadings, recalcitrant organic chemicals, wastewater treatment

## Abstract

The discharge of wastewater having recalcitrant chemical compositions can have significant and adverse environmental effects. The present study investigates the application of a catalytic ozonation treatment for the removal of recalcitrant organic chemicals (ROCs) from the water. Novel catalytic materials using vanadium (V) oxides deposited onto the surface of NaZSM-5 zeolites (V/ZSM) were found to be highly efficient for this purpose. The highly-dispersed V oxides (V^4+^ and V^5+^) and Si-OH-Al framework structures were determined to promote the surface reaction and generation of hydroxyl radicals. The constructed V1/ZSM_450_ (0.7 wt% of V loading and 450°C of calcination) exhibited the highest activity among the developed catalyst compositions. The V1/ZSM_450_-COP increased the mineralization rate of nitrobenzene and benzoic acid by 50 and 41% in comparison to single ozonation. This study demonstrates the enhanced potential of V/ZSM catalysts used with catalytic ozonation process (COP) for the treatment of chemical wastewaters.

## Introduction

Chemical wastewaters generated during the industrial production of dyes, perfumes, pesticides, and pharmaceuticals pose a significant threat to the environment and human health (Qu et al., 2007; Ao et al., 2019). Chemicals found in these wastewaters, such as nitrobenzene and benzoic acid, are environmentally persistent, have poor biodegradability and are highly toxic (Mantis et al., 2005). Development of efficient technologies for the removal of these recalcitrant organic chemicals (ROCs) is needed (Güm and Akbal, 2017; Nawaz et al., 2017). The application of catalytic ozonation process (COP) has been investigated for the development of an efficient method for the removal of ROCs from water (Li et al., 2017). The catalysts in the process facilitate the decomposition of ozone into more active species such as free radicals, and/or for the adsorption of chemicals that can react with dissolved ozone (Dong et al., 2008; Chen et al., 2018).

A wide variety of zeolites have been used as catalysts in COP. Important considerations for the zeolite catalysts include hydrophilicity/hydrophobicity, silica to alumina ratio (SiO_2_/Al_2_O_3_), surface area, and pore size (Amin et al., 2010; Jeirani and Soltan, 2017). The Y zeolites have been found to remove phenol and reduce chemical oxygen demand (COD) in solution, mainly via hydroxyl radicals (∙OHs)-mediated oxidation (Dong et al., 2008). The 4A zeolites enhanced the removal of paracetamol from water through an adsorption-mediated mechanism (Ikhlaq et al., 2018). The LTA zeolites improve the generation of •OHs from ozone, promoting total organic carbon (TOC) removal from a 2, 4-dimethylphenol solution (Vittenet et al., 2014). The presence of transition metallic oxides in zeolites can catalyze the decomposition of ozone into highly active radicals, enhancing oxidation (Valdés et al., 2009). For the MCM-41 zeolites, the loading of manganese (Mn), iron (Fe), or cerium (Ce) oxides increases the Lewis acid sites and surface hydroxyl groups (-OHs). The metals initiate ozone decomposition and generate •OHs that have been shown to mineralize nitrobenzene and *p*-chlorobenzoic acid (Bing et al., 2013). It is reported that Mn oxides loaded on SBA-15 zeolites significantly enhance mineralization of norfloxacin in COP (Sun et al., 2014). The protonated surface-OHs are the main reaction sites and promote •OHs generation (Chen et al., 2018). Vanadium (V) oxides are also efficient catalysts in COP and the support properties also influence the conversion and selectivity of the ROC (Chetty et al., 2012). To our knowledge, V oxides loaded zeolites have not been developed for utilization in COP.

ZSM-5 zeolites as typical catalysts are widely used during fluid catalytic cracking due to a high activity, appropriate acid strength, regular pore structure, and good hydrothermal stability (Martínez and Corma, 2011). The potential use of ZSM-5 zeolites as catalysts in COP has recently been reported (Chen et al., 2007). High silica ZSM-5 zeolites significantly enhanced the removal of COD and TOC of dimethyl phthalate solution in an ozone/ultraviolet process via •OHs oxidation (Chen et al., 2007). The ZSM-5 zeolite is a highly adsorbent, providing a large reaction surface area between ozone and phenol in water (Amin et al., 2010). Ozonation aided by ZSM-5 zeolites is speculated to be a non-radical mechanism. The ZSM-5 zeolites functions as ozone reservoirs and adsorbents of organic chemicals. This activity is dependent upon the ratio between SiO_2_ and Al_2_O_3_ or hydrophobicity rather than acidity or counter ions (Ikhlaq and Kasprzyk-Hordern, 2017). Our previous studies further determined that both NaZSM-5 and adsorption-saturated HZSM5 zeolites follow •OHs-mediated oxidation in COP of nitrobenzene. Surface Si-O bonds and/or Si-O(H)-Al structures are the active sites of the ZSM-5 zeolites. The loading of Ce, Fe, or Mn oxides increase the catalytic activity relative to ZSM-5 zeolites alone (Chen et al., 2018). Efficiencies and mechanisms of ROCs mineralization using ZSM-5 zeolites catalyzed ozonation are not yet clearly identified. The V oxides supported on metallic oxides or carbon materials have been previously utilized to increase the efficiency for the advanced oxidation treatments of ROCs (Maddila et al., 2014; Rivoira et al., 2017). The V oxides loaded ZSM-5 zeolites (V/ZSM) as catalysts in COP for mineralization of ROCs have not been investigated.

In the present study, NaZSM-5 zeolites were loaded with V oxides and characterized. Mn supported NaZSM-5 zeolite was used as the reference considering the high catalytic activity of in COPs (Chen et al., 2018). Insights into catalytic efficiencies and mechanisms of those zeolites in COP for mineralizing nitrobenzene and benzoic acid in water were investigated for its potential application.

## Experimental

### Materials

NaZSM-5(30) zeolite (sodium [Na] type of counter ion, SiO_2_/Al_2_O_3_ ratio at 30) was selected as a parent catalyst (PZSM) and was purchased from Shanghai Sunny Molecular Sieve Plant, Shanghai, China. Nitrobenzene (99.8 wt%), benzoic acid (99.5 wt%), ammonium metavanadate (NH_4_VO_3_, 99.95 wt%), oxalic acid (H_2_C_2_O_4_, 99.6 wt%), sodium hydroxide (NaOH, 96 wt%), hydrochloric acid (HCl, 38 wt%), sodium bicarbonate (NaHCO_3_, 99.5 wt%), manganese nitrate solution (Mn(NO_3_)_2_; 50 wt%) and disodium hydrogen phosphate (Na_2_HPO_4_, 99 wt%) were all obtained from Beijing Chemical Reagents Co., Ltd., Beijing, China. The ultrapure water (18.2 mΩ/cm) was produced by a Direct-Pure UP ultrapure water system (Rephile Shanghai Bioscience Co., Ltd., Shanghai, China).

### Catalyst Preparation

The V/ZSM catalysts were prepared according to the incipient wetness impregnation method. Varying amounts of NH_4_VO_3_ and 4 g of H_2_C_2_O_4_ were added to ~80 g of ultrapure water. The mixture was stirred for 30 min at 40°C to form a solution. Hundred gram of PZSM was saturated with the solution containing 2.4 or 4.0 g of NH_4_VO_3_. The impregnated samples were then calcinated at 450°C for 4 h in air after drying at 110°C for 12 h to yield V1/ZSM_450_ and V2/ZSM_450_, respectively. The calcination at 550°C was to yield V1/ZSM_550_ and V2/ZSM_550_. The referenced catalyst of Mn supported NaZSM-5 zeolite (Mn/ZSM) with 1% Mn loading (by XRF) was prepared by the incipient wetness impregnation. An amount of 0.67 g of 50 wt% Mn(NO_3_)_2_ solution was added with 3.0 g water followed by impregnation of 10 g NaZSM-5 with the above solution. The resulting material was dried at 90°C for 4 h and calcined at 450°C for 4 h.

### Characterization of the Catalysts

The surface morphology was examined on a Quanta 200F scanning electron microscope (SEM) and a Tecnai G2 F20 transmission electron microscope (TEM) (FEI, Hillsboro, OR, USA). The crystals were observed by X-ray powder diffraction (XRD) with an XRD-6000 powder diffraction instrument (Shimadzu, Kyoto, Japan). The functional groups on the surface were identified with a Magna-IR 560 ESP FT-IR spectrometer (Nicolet, Madison, WI, USA). The surface area and pore volume were determined with an ASAP 2000 accelerated surface area and porosimetry system (Micromeritics, Norcross, GA, USA). The composition was determined with a ZSX-100E X-ray fluorospectrometer (XRF) (Rigaku, Tokyo, Japan). Surface element distribution was recorded with a PHI Quantera SXM X-ray photoelectron spectrometer (XPS) (ULVAC-PHI, Chanhassen, MN, USA). The pH of point of zero charges (pH_pzc_) of catalysts was determined according to the pH-drift procedure (El-Bahy et al., 2008).

### Ozonation of Recalcitrant Organic Chemicals

Nitrobenzene and benzoic acid are typical ROCs found in chemical wastewaters (Chen et al., 2018; Suryavanshi et al., 2018). In this study, nitrobenzene, and benzoic acid were utilized as model ROCs to investigate efficiencies and mechanisms of V/ZSM catalyzed ozonation (V/ZSM-COP). Nitrobenzene solution and benzoic acid solution were prepared with a concentration of 100 mg/L in ultrapure water. The initial TOC and pH values of nitrobenzene solution and benzoic acid solution were 55.9 or 67.5 mg/L and 6.5 or 3.5, respectively.

The experimental ozonation system was constructed using a 40L of oxygen tank (Shandong Tianhai High Pressure Container Co., Ltd., Linyi, China), a COM-AD-02 ozone generator (Anseros Asvanced Oxidation Technologies Co., Ltd., Tübingen-Hirschau, Germany), two GM-6000-OEM ozone gas analyzers (Anseros Asvanced Oxidation Technologies Co., Ltd., Tübingen-Hirschau, Germany), a D07-7 mass flow controller (Beijing Sevenstar Flow Co., Ltd., Beijing, China), a D08-1F flow readout box (Beijing Sevenstar Flow Co., Ltd., Beijing, China), a 1,000 mL quartz column reactor and an exhaust gas collector. The reactor was placed on a ZNCL-BS intelligent magnetic stirrer (Shanghai Kankun Instrument Equipment Co., Ltd., Shanghai, China) at 800 rpm to promote the mass transfer between ROCs, ozone and catalysts.

During ozonation experiments, an aliquot of 500 mL of nitrobenzene solution or benzoic acid solution and 0.5 g of catalyst was added into the reactor at 25°C. The gaseous ozone was then introduced through a porous diffuser at the bottom of the reactor having a flow rate of 20 mg min^−1^. An aliquot of 10 mL of treated solution was extracted into a 20 mL of syringe when sampling. The solution in the syringe was then pressed through a 0.45 μm end filter (Tianjin Jinteng Experimental Equipment Co., Ltd., Tianjin, China) to remove catalyst particles before further analysis. Adsorption experiments and single ozonation (SOP) were performed with the same experimental system as the COP. The initial pH values of solutions were adjusted with 1 N NaOH or HCl. Experiments were performed in triplicate.

The reaction rate constants (*k*_*m*_) of mineralization of nitrobenzene or benzoic acid in ozonation were calculated with equations (1) (Portela et al., 2001).

(1) $$\begin{array}{r} \ln\left(\frac{TOC_{0}}{TOC}\right)=k_{m}t \end{array}$$

Where, TOC_0_, and TOC represent the TOC value of nitrobenzene solution and benzoic acid solution before and after ozonation. The •OH quenching experiments were performed to identify the oxidation mechanism. As an •OH scavenger, NaHCO_3_ was added into the solution at various concentrations prior to ozonation experiments.

The TOC and pH values of nitrobenzene solution and benzoic acid solution were measured on a TOC-L CPH CN 200 TOC analyzer (Shimadzu, Kyoto, Japan) and an MP 220 pH meter (Mettler Toledo, Greisensee, Switzerland). Degradation products were analyzed by a 7890B gas chromatograph-5977B mass selective detector (GC-MS) (Agilent, Santa Clara, CA, USA) with a 30 m × 0.25 μm × 0.25 mm DB-35 GC column. The sample pretreatment process was as follows: A 6 mL of solid-phase extraction cartridge (Sep-pak C18, 1 g, Waters, Milford, MA, USA) was conditioned by rinsing with 10 mL of methanol followed by 10 mL of acidified ultrapure water (pH value at 2). The samples were first filtered through a 0.45 μm Supor membrane filter (PALL, Ann Arbor, MI, USA) before pretreatment. A 10 mL of filtered sample was then pumped into the cartridge at 5 mL/min. The cartridge was rinsed with 20 mL of acidified ultrapure water (pH value at 2) to remove salts. The cartridge was then eluted with 10 mL of methanol. The filtrate was concentrated to 1 mL using nitrogen blowing prior to GC-MS analysis. The initial GC column temperature was remained at 65°C for 4 min, ramped to 210°C at 5°C/ min and then kept for 5 min. The ultra-pure helium was used as the carrier gas at 1.4 mL min^−1^. One microliter of concentrated sample was injected into the GC column using a splitless injector with a column head pressure of 10 psi (69 kPa).

## Results and Discussion

### Characteristics of V/ZSM Catalysts

**Figure 1:**
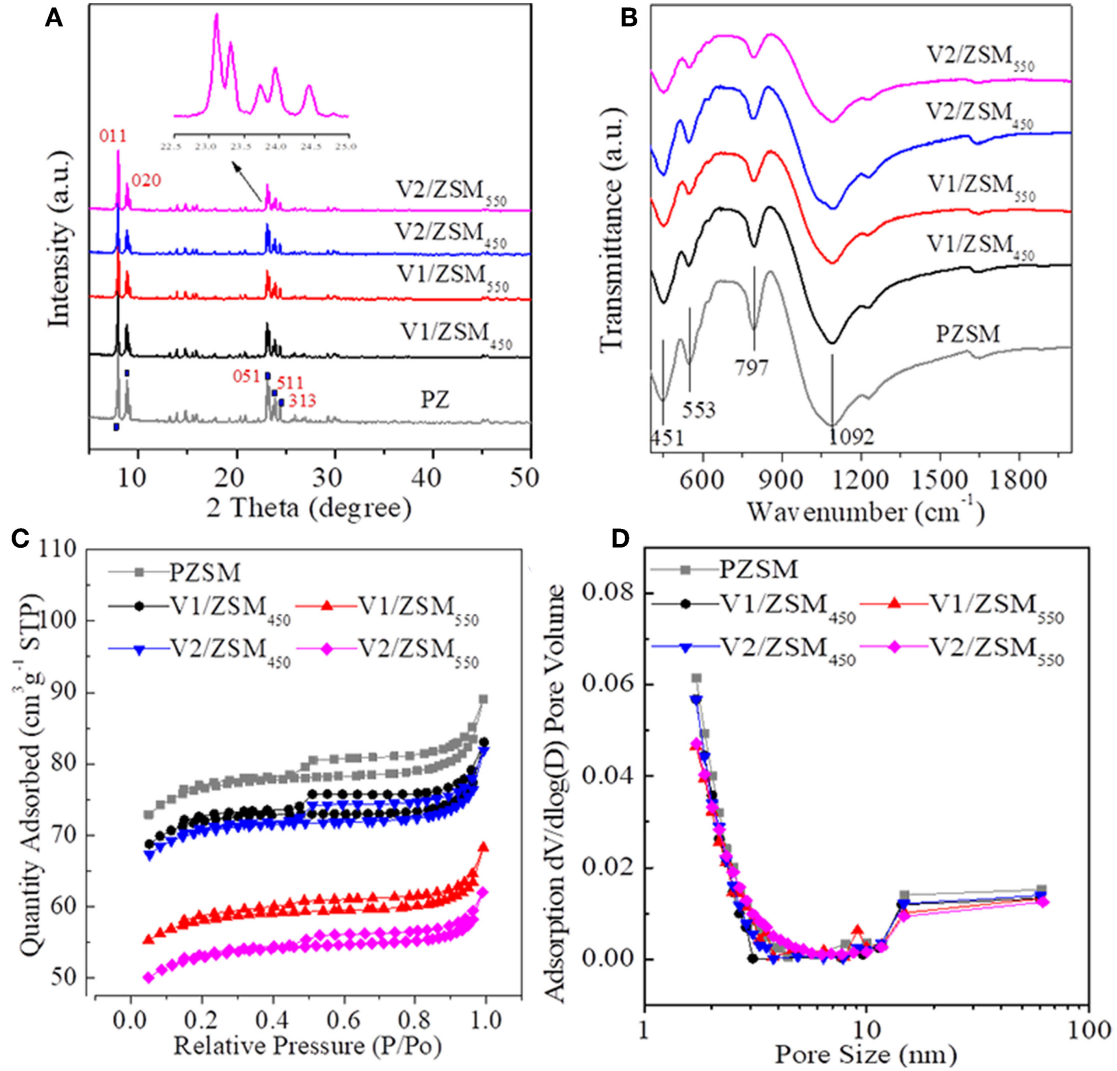
XRD spectra **(A)**, FT-IR **(B)** spectra, adsorption-desorption isotherms **(C)**, and pore distributions **(D)** of ZSM-5 zeolites.

**Figure 2:**
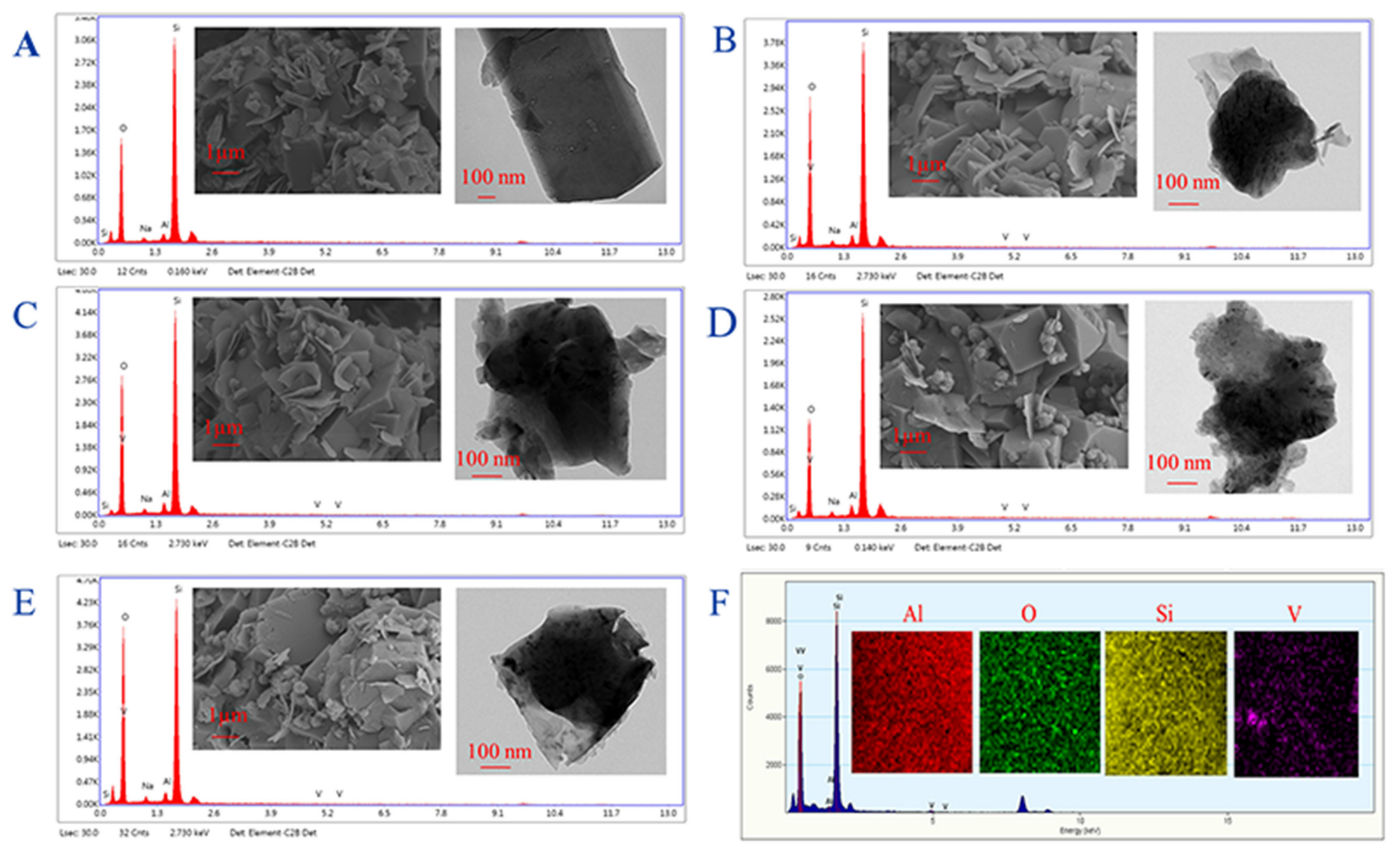
SEM and TEM images (insert) and EDX spectra of ZSM-5 zeolites: PZSM **(A)**, V1/ZSM_450_ **(B)**, V1/ZSM_550_ **(C)**, V2/ZSM_450_ **(D)**, and V2/ZSM_550_ **(E)** and element mapping-EDS spectra of V1/ZSM_450_ **(F)**.

The V/ZSM catalysts mainly exhibited XRD patterns at 2 theta of 7.86°, 8.78°, 23.18°, 23.90°, and 24.40°, which are characteristic of crystal face diffraction of (011), (020), (051), (511), and (313), respectively. The XRD patterns of V/ZSM catalysts were similar to that of PZSM (Figure 1A). The peak intensities are however slightly decreased when compared with PZSM. The crystallinity of V1/ZSM_450_, V2/ZSM_450_, V1/ZSM_550_, and V2/ZSM_550_, is 85, 77, 80, and 73%, relative to PZSM (100%), respectively. A high calcination temperature and V loading can damage and overlay part of the ZSM-5 framework, reducing crystallinity. No prominent XRD diffraction peaks from V oxides are observed due to the high dispersion and low concentration (El-Bahy et al., 2008). The FT-IR spectra in a range of 450-2000 cm^−1^ for PZSM and V/ZSM catalysts are displayed in Figure 1B. The peaks at 451, 553, 797, and 1,092 cm^−1^ are ascribed to the ZSM-5 framework (Zhao et al., 2015). Introduction of V oxides does not change the position of FT-IR peaks, however, decreases the intensity of characteristic peaks, suggesting a light collapse of the structure and/or the presence of V oxides on the surface. These results are in good accordance with the XRD analysis. PZSM and V/ZSM catalysts all are IV type of isotherms and show well-developed microporous structures (Figures 1C,D). The increase of calcination temperature and V loading decreases the surface area of V/ZSM catalysts (Table 1). Surface morphologies of V/ZSM catalysts remain unchanged by V loading (Figure 2). Irregular shapes of V oxides can be observed on the surface of V/ZSM catalysts by TEM (Figure 2). Further mapping focusing on V1/ZSM_450_ confirmed that V is uniformly distributed for the V1/ZSM_450_ (Figure 2F). The V contents determined by EDX are lower than those from XRF, suggesting a high bulk deposition (Table 1).

**Table 1:** Surface areas and pore structures of ZSM-5 zeolites by N_2_ adsorption-desorption and V content by EDX and XRF.

**Catalysts**	**Texture properties by N**_****2****_ **adsorption-desorption**				**V contents (wt%) by**	
	**Specific surface areas m^**2**^g^**−1**^**	**Micropore surface areas m^**2**^g^**−1**^**	**Total volumes cm^**3**^g^**−1**^**	**Micropore volumes cm^**3**^g^**−1**^**	**XRF**	**EDX**
PZSM	248	213	0.14	0.10	–	–
V1/ZSM_450_	232	202	0.13	0.10	1.0	0.44
V1/ZSM_550_	188	161	0.10	0.08	0.98	0.31
V2/ZSM_450_	229	200	0.13	0.10	1.72	0.77
V2/ZSM_550_	172	144	0.09	0.07	1.69	0.65

**Figure 3:**
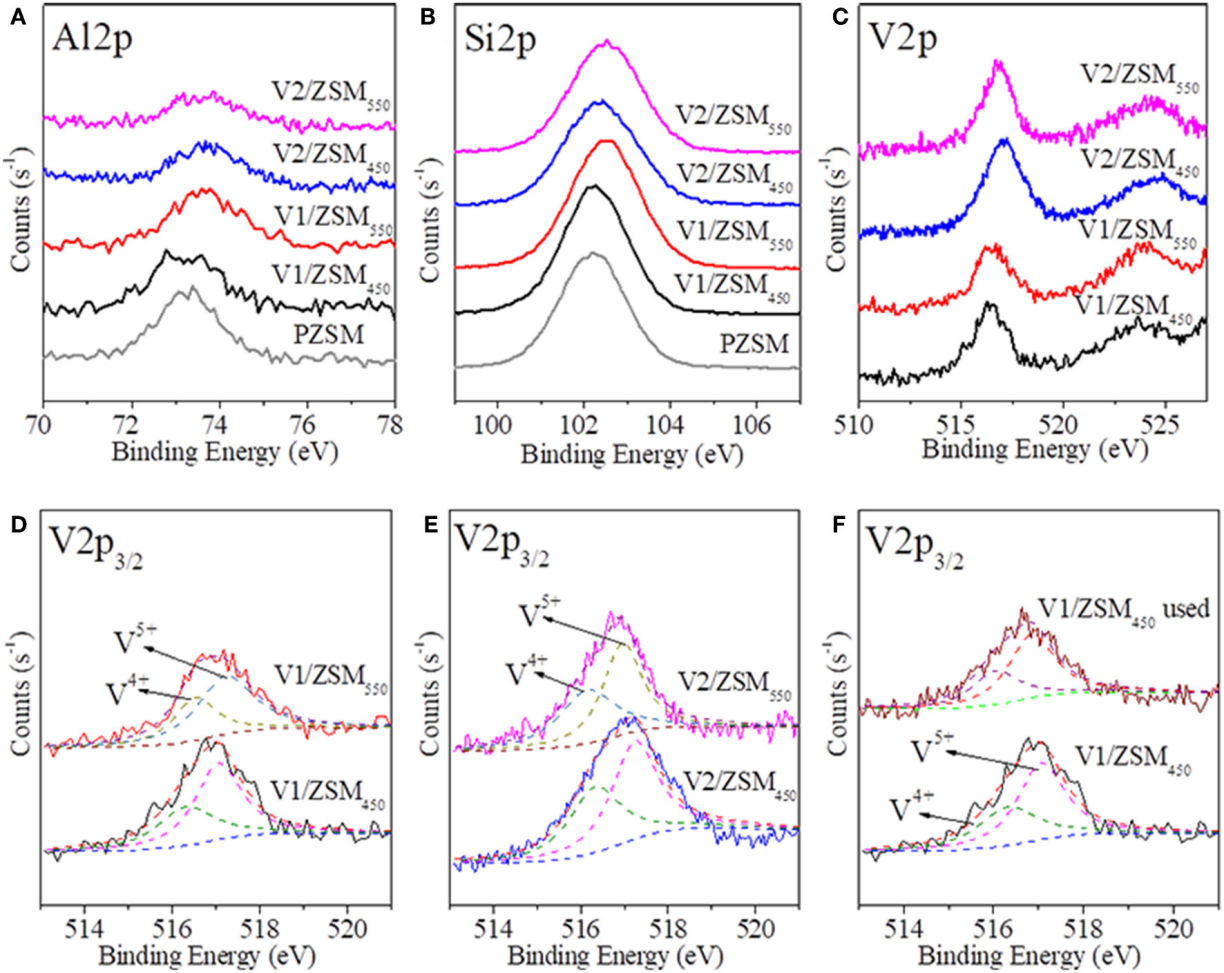
XPS spectra of Al2p **(A)**, Si2p **(B)**, and V2p **(C–F)** for ZSM-5 zeolites.

XPS spectra of Al2p, Si2p, and V2p of PZSM and V/ZSM catalysts are shown in Figure 3. The binding energies of Al2p at 74 eV and Si2p at 102 eV are, respectively attributed to Al^3+^ and Si^4+^ oxides from Si-O-Al frameworks (Hadnadjev et al., 2008; Plymale et al., 2015) (Figures 3A,B). Surface Si and Al oxides of V/ZSM catalysts slightly decrease compared to PZSM due to the loading of V oxides. More V oxides are loaded onto the surface of V2/ZSM_450_ and V2/ZSM_550_, compared to V1/ZSM_450_ and V1/ZSM_550_ according to the peak intensity of V2p spectra (Figure 3C). The XPS spectra from V2p mainly center at the ranges of 515–518 and 523–526 eV. The former was assigned to V2p_3/2_ (V^5+^) and V2p_3/2_ (V^4+^) oxides at 517.2 and 516.3 eV, and the latter associated with V2p_1/2_ (V^5+^) and V2p_1/2_ (V^4+^) oxides at 523.4 and 524.5 eV (Silversmit et al., 2004) (Figures 3D,E). The molar ratio of V^5+^ to V^4+^ (V^5+^/V^4+^ ratio) of V1/ZSM_450_, V2/ZSM_450_, V1/ZSM_550_, and V2/ZSM_550_ was 1.59, 1.25, 1.68, and 1.23, respectively, according to V2p_3/2_ peak fitting results. Overall, the V was found loaded on the surface of V/ZSM catalysts in forms of V^5+^ and V^4+^ oxides. The V loading or a high calcination temperature might result in coverage and partial collapse of the Si-O-Al frameworks. The V^5+^/V^4+^ ratio of used V1/ZSM_450_ was 1.82, higher than that (1.59) of parent V1/ZSM_450_. It suggests partial metal V^4+^ was oxidized to V^5+^. Besides, the peak intensity of V2p for V1/ZSM_450_ was decreased after COP, due to metal V loss.

### Removal Efficiencies of ZSM-5 Zeolites Catalyzed Ozonation

#### TOC Removals of Adsorption SOP and COPs

**Figure 4:**
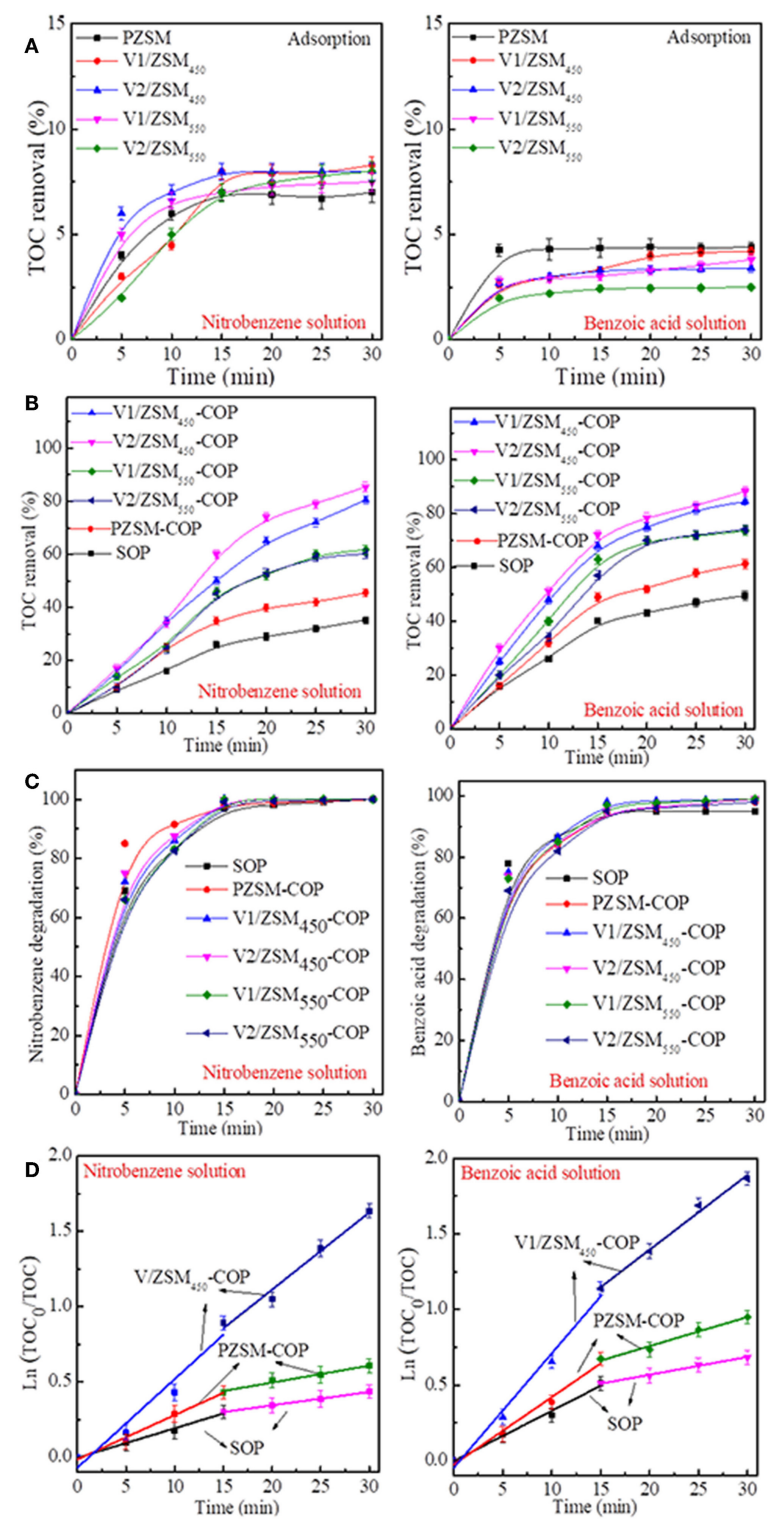
TOC removal of nitrobenzene solution and benzoic acid solution during adsorption **(A)**, SOP and COPs **(B)**; concentration changes of nitrobenzene solution and benzoic acid solution upon SOP and COPs **(C)** using various ZSM-5 zeolites; Pseudo-first-order kinetics plots of SOP, PZSM-COP and V1/ZSM450-COP for nitrobenzene solution and benzoic acid solution **(D)**. Note: Initial pH values of the nitrobenzene solution and benzoic acid solution are 6.5 and 3.5. Initial pH values of the nitrobenzene solution and benzoic acid solution are 6.5 and 3.5.

The PZSM and V/ZSM catalysts exhibit weak adsorption toward both nitrobenzene and benzoic acid in water. However, there was a significant difference in saturation time and TOC removal and between different chemicals. The adsorption reached saturation in 30 min for the nitrobenzene solution and in 15 min for the benzoic acid solution. PZSM and V/ZSM catalysts removed more TOC from the nitrobenzene solution (7.0–8.3%) compared to the benzoic acid solution (2.5–4.4%) within 30 min (Figure 4A). Nitrobenzene is polar relative to benzoic acid, therefore, is absorbed by the weakly hydrophobic NaZSM-5 (30) zeolite (Nakamoto and Takahashi, 1982). The loading of V oxides and calcination temperature used in this study had a negligible impact on the adsorption capacity of V/ZSM catalysts. Adsorption is more significantly influenced by the chemical nature of the compounds in the water, when compared with the effects from the catalyst surface area and pore volume (Table 1). SOP removes 35.3 and 49.5% of TOC from the nitrobenzene solution and benzoic acid solution after 30 min treatment (Figure 4B). COPs using ZSM-5 zeolites remove 45.5–85.4% and 61.3–88.3% of the TOC from the nitrobenzene solution and benzoic acid solution after 30 min, respectively (Figure 4B). TOC removal upon application of PZSM-COP and V/ZSM-COPs are higher than the sums of their adsorption and SOP, confirming the catalytic activity of ZSM-5 zeolites. The dominant TOC removal during COPs using ZSM-5 zeolites results from ozonation rather than adsorption. PZSM-COP increased TOC removal of nitrobenzene from solution by 10.2% and benzoic acid from solution by 11.8% when compared to SOP. The degradation of nitrobenzene (99–100%) and benzoic acid (95–99%) in solution were identical for both the COPs and SOP (Figure 4C) during the 30 min treatment. The concentration changes for the nitrobenzene and benzoic acid solution (Figure 4C) are different as determine by the changes in TOC (Figure 4B) during both SOP and COPs. Smaller molecular intermediates are initially generated during the oxidation of nitrobenzene and benzoic acid; COPs can effectively further mineralize these species, while SOP is usually ineffective.

The frameworks of PZSM are relevant to the catalytically active sites, promoting the mineralization of chemicals in COP. Acidic sites are usually thought to be active on the zeolites (Saeid et al., 2018). The Si-ONa-Al structure may be exchanged to Si-OH-Al (Shirazi et al., 2008), as an active site for ZSM-5 zeolites. The V/ZSM-COPs increase TOC removal of nitrobenzene solution by 14.7–39.9% and benzoic acid solution by 12.3–27.0% in comparison with PZSM-COP. These differences indicate that by the loading of V oxides on PZSM the COP is improved. Surface distributed V oxides (V^4+^ and V^5+^) function as major active sites of V/ZSM catalysts. Excessive loading of V oxides does not noticeably improve the TOC removal during V/ZSM-COPs. Even though the loading of V oxide is doubled, the TOC removal of V2/ZSM_450_ (85.4% for nitrobenzene and 88.3% for benzoic acid) was a little higher than that of V1/ZSM_450_ (80.5% for nitrobenzene and 84.5% for benzoic acid). The V2/ZSM_550_ (60.2% for nitrobenzene and 74.2% for benzoic acid) and V1/ZSM_550_ (61.7% for nitrobenzene and 73.6% for benzoic acid) have similar results. The excessive loading of metallic oxides may reduce active sites, weakening interactions between metallic oxides, and supports (Chen et al., 2017a). In this application, excessive V oxides may partially block Si-OH-Al structures, reducing activity. The increased activity from the active V oxides is partially offset by decreased Si-OH-Al sites. The V1/ZSM_450_ and V2/ZSM_450_ exhibit high TOC removal relative to V1/ZSM_550_ and V2/ZSM_550_. A high calcination temperature can decrease the catalytic activity of V/ZSM catalysts (Chen et al., 2017b). The V/ZSM catalysts that are calcinated at 550°C show lower crystallinity and surface areas than those at 450°C, suggesting the loss of Si-OH-Al structures. The co-effect of various active sites coexisting on the support favors catalytic activity (Tu et al., 2012). For V/ZSM catalysts, its activity is dependent upon two kinds of active sites that coexist and interact cooperatively, the V oxides (V^4+^ and V^5+^) and Si-OH-Al structures. The cooperative effects from V oxides and Si-OH-Al structures on V/ZSM catalysts are however reduced by high temperature calcination, resulting in lowered catalytic activity for both V1/ZSM_550_ and V2/ZSM_550_. Among the V/ZSM catalysts, V1/ZSM_450_ shows the high catalytic activity for the COP removal of nitrobenzene and benzoic acid in solution. By contrast, the TOC removal of the referenced Mn/ZSM (72.2% for nitrobenzene and 75.4% for benzoic acid) was a little lower than that of V1/ZSM_450_ (80.5% for nitrobenzene and 84.5% for benzoic acid). The result further reveals the advantage of V1/ZSM_450_. The remainder of this study is focused on PZSM and V1/ZSM_450_.

### Mineralization Kinetics of SOP and COPs

The mineralization of nitrobenzene and benzoic acid both follow pseudo-first-order kinetics during SOP, PZSM-COP and V1/ZSM_450_-COP treatments that occur in two phases at 0–15 and 15–30 min, respectively (Figure 4D). For the nitrobenzene solution, *k*_m(0−15min)_ values are 0.0197, 0.0295, and 0.0471 min^−1^, *k*_m(15−30min)_ values are 0.0891, 0.0112, and 0.061 min^−1^, over SOP, PZSM-COP, and V1/ZSM_450_-COP, respectively. For benzoic acid solution, *k*_m(0−15min)_ are 0.0332, 0.0446, and 0.0757 min^−1^, *k*_m(15−30min)_ values are 0.0118, 0.0192, and 0.0496 min^−1^ over SOP, PZSM-COP and V1/ZSM_450_-COP. PZSM-COP shows high *k*_m_ values relative to SOP, verifying the catalytic activity of PZSM. V1/ZSM_450_-COP has high *k*_m_ values compared to PZSM-COP and SOP, demonstrating the powerful catalytic activity of V1/ZSM_450_. The *k*_m_ values of nitrobenzene are lower than those of benzoic acid during SOP, PZSM-COP, and V1/ZSM_450_-COP, reflecting the ease of benzoic acid mineralization when compared with nitrobenzene. The *k*_m(15−30min)_ values are lower than the *k*_m(0−15min)_ values, suggesting that various mineralization mechanisms are involved. The reaction intermediates produced in solution add complexity toward understanding the mineralization process.

### Removal Mechanisms of ZSM-5 Zeolites Catalyzed Ozonation

#### Changes of Solution pH Values in Adsorption, SOP, and COPs

**Figure 5:**
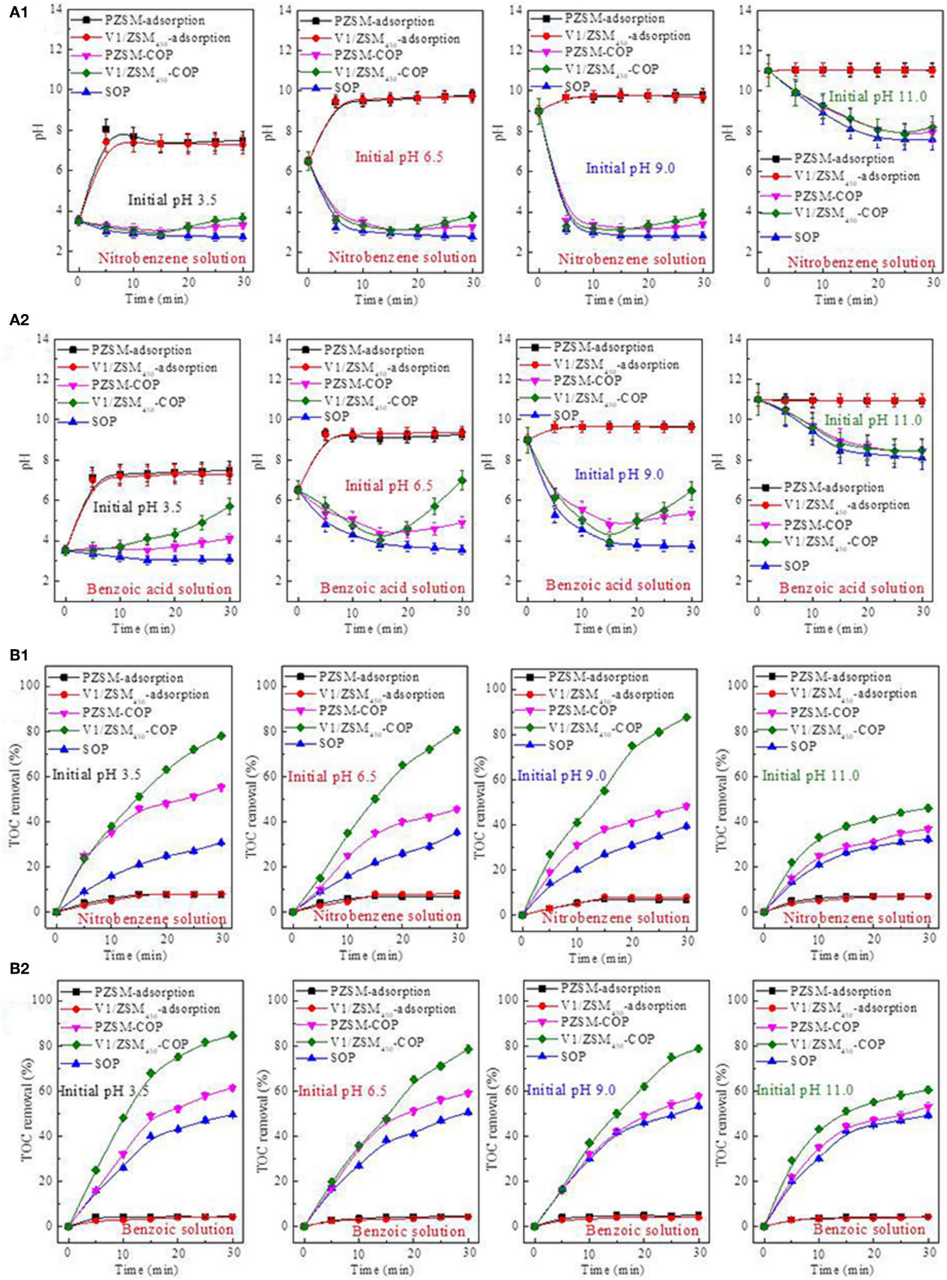
pH value changes of the nitrobenzene solution **(A1)** and benzoic acid solution **(A2)** during adsorption, SOP, PZSM-COP and V1/ZSM_450_-COP; Influence of the initial pH values on TOC removal of nitrobenzene solution **(B1)** and benzoic acid solution **(B2)** during adsorption, SOP, PZSM-COP, and V1/ZSM_450_-COP.

The changes in wastewater pH during the adsorption and ozonation treatments were measured for mechanistic insights. The pH values for the nitrobenzene (Figure 5A1) and benzoic acid solutions (Figure 5A2) rapidly increased during PZSM and V1/ZSM_450_ adsorption under acidic and nearly neutral conditions (initial pH values at 3.5 and 6.5) (Figure 5A). The increase in pH values result from the exchange between H^+^ in solutions and the Na^+^ component of the ZSM-5 zeolites. Furthermore, the original Si-ONa-Al structures of the ZSM-5 zeolite are partly changed to Si-OH-Al structures. The observed changes in pH resulting from the PZSM and V1/ZSM_450_ adsorptions under alkaline conditions (initial pH values at 9 and 11) (Figure 5A), demonstrate that ion exchange did not occur. The two solutions both show a continuous pH decline during SOP treatment. However, this declining tendency is differentiated depending upon the initial pH value (Figure 5A). The pH values both exhibit downward trends under highly acidic (initial pH value at 3.5) and alkaline (initial pH value at 11) conditions. They all display rapid decreases during the initial 5 min, then slowly decrease under weak acidity (initial pH value at 6.5) and alkaline (initial pH value at 9) conditions (Figure 5A). The extent of this decrease is less than those that occur under weakly acidic and alkaline conditions. The SOP produces acidic intermediates from the chemical degradation resulting in the decrease of solution pH values (Zhao et al., 2008). The SOP functions mainly via direct oxidation and only a small amount of •OHs is involved with oxidation. Limited •OHs generation occurs at low pH values, and the •OHs are apt to dissociation at very high pH values (Beltrán et al., 1998), lowering the production rate of acidic intermediates. Therefore, the pH values only exhibit moderate reductions. Under weakly acidic and alkaline conditions ozone decomposition is the dominant process resulting in the generation of •OHs (Elovitz et al., 2008). The production of acidic intermediates results in the rapid decline in pH values. The acidic solutions inhibit •OHs generation, also resulting in decreased acidic intermediate production.

The changes in pH value during the SOP, PZSM-COP, and V1/ZSM_450_-COP treatments are similar when performed under highly alkaline conditions (initial pH value at 11). The SOP, PZSM-COP, and V1/ZSM_450_-COP follow direct oxidation mechanisms. The catalytic activity of the ZSM-5 zeolites is not significant in solutions having high initial pH values (11). Solutions ranging from strong acidity (initial pH value at 3.5) to weakly alkaline (initial pH value at 9), treated using PZSM-COP and V1/ZSM_450_-COP both exhibit decreased pH during the initial 15 min followed by an increase (Figure 5). The increased pH observed after 15 min implies that the acidic intermediates are being mineralized. The mineralization is usually associated with the generation of reactive oxidizing species (e.g., •OHs) or chemisorption at the surface (Andreozzi et al., 1996; Ikhlaq et al., 2018). The greater extent of the pH increase from V1/ZSM_450_-COP compared with PZSM-COP suggests a stronger mineralization effect (Figure 5A). At the same initial pH value, the increase in pH values for nitrobenzene is smaller than that with the benzoic acid solution (Figure 5A) demonstrating that the intermediates of benzoic acid are mineralized easily compared with those from nitrobenzene.

#### Influences of Initial pH Values on TOC Removals of Adsorption, SOP, and COPs

The initial pH values for the nitrobenzene and benzoic acid solutions had no impact on the adsorption capacity of PZSM and V1/ZSM_450_ (Figure 5B). They both minimally adsorbed nitrobenzene (Figure 5B1) and benzoic acid (Figure 5B2) from solution at various initial pH values. The initial pH values did however significantly influence the efficiencies of SOP, PZSM-COP, and V1/ZSM_450_-COP (Figure 5B). An appropriate concentration of hydroxide in solution may further promote ozone decomposition into •OHs and improve mineralization (Elovitz et al., 2008). Therefore, the SOP efficiently removed TOC removal under weak alkaline (initial pH value at 9) condition compared to highly acidic (initial pH value at 3.5) and alkaline (initial pH value at 11).

The PZSM-COP and V1/ZSM_450_-COP more efficiently removed TOC relative to SOP at initial pH values between 3.5 and 9. The TOC removal at acidic (initial pH value at 3.5) conditions using PZSM-COP was higher than those performed under weak acidity (initial pH value at 6.5) and weak alkalinity (initial pH value at 9). Under acidic conditions, the Na^+^ in the Si-ONa-Al structures of ZSM-5 zeolites interacts with the H^+^ in solution. The increased pH values of solutions during PZSM adsorption confirm the presence of this ion exchanges mechanism. The newly formed Si-OH-Al structures increase the acidity of ZSM-5 zeolites, enhancing the catalytic activity. The V1/ZSM_450_-COP has high TOC removals relative to PZSM-COP under the initial pH values from between 3.5 and 9. Efficient TOC removal for the different chemicals occurs at different initial pH values.

Using the V1/ZSM_450_-COP the highest TOC removed from the nitrobenzene solution (Figure 5B1) occurred at an initial pH value of 9, which is close to the pH_pzc_ value (9.25) of V1/ZSM_450_. Uncharged surface hydroxyl groups (-OHs) are beneficial for ozone decomposition into •OHs (Zhao et al., 2008). The surface -OHs on the V oxides (V-OHs) have a neutral charge at an alkaline pH value of 9, this increases the interactions that occur between the surface V-OHs and ozone. When the initial pH values are lower than the pH_pzc_ value of V1/ZSM_450_, a portion of surface -OHs on the V oxides are protonated (V-OH_2_^+^) and the interaction between V-OHs and ozone is reduced. This effect can be clearly observed from the experimental data generated at the lower 3.5 pH. The results are further influenced by the simultaneous formation of Si-OH-Al that occurs under acidic conditions, increasing the catalytic activity. Because of this, the use of V1/ZSM_450_-COP results in nearly identical TOC removals that are observed at the pH values of 3.5 and 6.5. The pKa value of nitrobenzene in water is 11.9, which is greater than the experimentally used initial pH values. The high pKa values and at lower operational pHs conditions mean that the nitrobenzene is not dissociated and exists in its molecular form (Langer et al., 2008). The surface catalytic oxidation of nitrobenzene is not significantly influenced by initial pH, under our experimental conditions.

Using the V1/ZSM_450_-COP for the treatment of benzoic acid (Figure 5B2), the interaction between surface V-OHs and ozone are reduced at the initial pH value of 6.5, relative to 9. At the pH values of 6.5 and 9, benzoic acid exists mainly in an ionic state due to its pKa value of 4.2.The protonated V-OH_2_^+^ on the catalyst surface facilitates the interactions with benzoate at the pH value of 6.5, increasing the surface oxidation reactions with benzoate compared to a pH value of 9. The V1/ZSM_450_-COP similarly removed TOC at the pH values of 6.5 and 9. Due to its pKa, benzoic acid exists in its molecular state at pH 3.5, reducing surface oxidation reactions between V-OH_2_^+^ and benzoate. The active catalytic sites on Si-OH-Al are also simultaneously increased, promoting oxidation.

At an initial pH value of 11 the SOP and PZSM-COP comparably removed TOC. At this pH, the catalytic activity of ZSM-5 zeolites is inhibited. Under strong alkaline conditions, Si-ONa-Al structures are dominant when compared with Si-OH-Al. Therefore, the Si-OH-Al derived catalytic activity does not occur with PZSM and V1/ZSM_450_. At high pH values above the pH_pzc_ (9.25) for V1/ZSM_450_, a portion of surface -OHs on the V oxides are presented in a deprotonated form of V-O^−^. The catalytic activity from the V-OHs and ozone interaction declines for V1/ZSM_450_. The V1/ZSM_450_-COP exhibits low TOC removal at pH value of 11.

#### Removal Mechanisms of Chemicals in ZSM-5 Zeolites Catalyzed Ozonation

**Figure 6:**
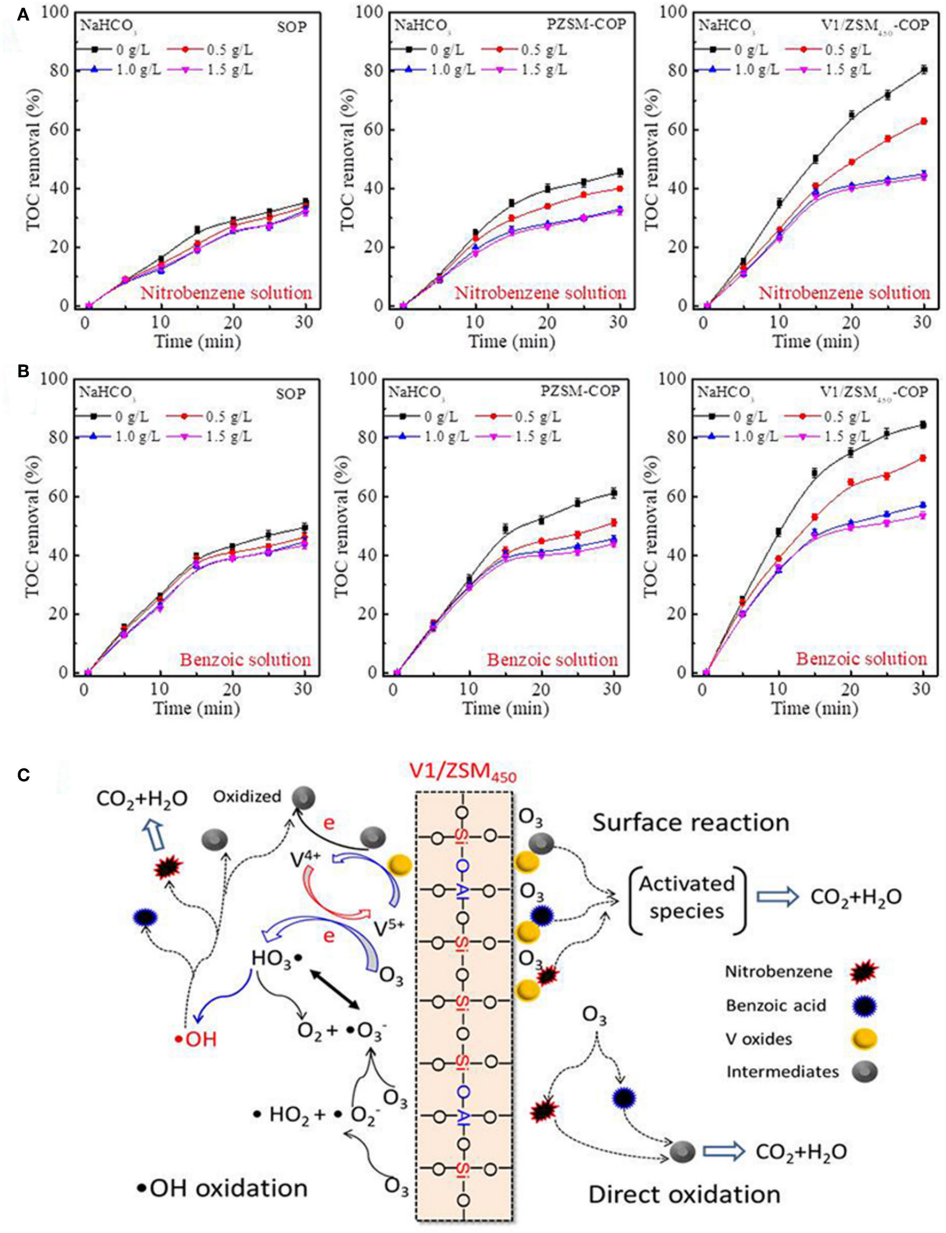
Influences of NaHCO_3_ addition on the TOC removal of nitrobenzene solution **(A)** and benzoic acid solution **(B)** during SOP, PZSM-COP and V1/ZSM_450_-COP; proposed mechanism for nitrobenzene and benzoic acid removal with V1/ZSM_450_-COP **(C)**. Initial pH values of the nitrobenzene solution and benzoic acid solution are 6.5 and 3.5.

During the COP the generation of •OHs are promoted by various catalysts and can facilitate the removal of ROC (Chen et al., 2018). During SOP, the introduction of NaHCO_3_ has an influence on the TOC removed from the nitrobenzene and benzoic acid containing solutions (Figures 6A,B). The SOP process removes the TOC mainly by direct oxidation and •OHs are not significantly generated. The TOC removed by PZSM-COP are nearly equal to the SOP after the addition of 0.5 and 1.0 g/L NaHCO_3_ (Figure 6A). The mineralization that occurs during the PZSM-COP is the result from both •OHs mediated oxidation and direct oxidation. The addition of NaHCO_3_ significantly inhibits the V/ZSM_450_-COPs activities relative to PZSM-COP (Figures 6A,B), suggesting that the loaded V oxides increases activity by generating •OHs. Electron transfers between multivalent metallic oxides and ozone molecules accelerate ozone decomposition into active •OHs (Zhuang et al., 2014). Co-existing metal V oxides (V^5+^ and V^4+^) further promote •OHs generation via electron transfer. Further inhibition of the V/ZSM_450_-COPs activity was not observed at a higher concentration of NaHCO_3_, up to 1.5 g/L. The V/ZSM_450_-COPs still exhibited high TOC removal rates relative to SOP (Figures 6A,B), suggesting that •OHs oxidation is not the only mineralization process. It was previously determined that the degradation of chemicals in COPs can be enhanced by the formation of metallic oxides-chemicals complexes, such as MnO_2_-oxalic acid (Andreozzi et al., 1996) or CoO-carboxylic acid (Pines and Reckhow, 2003), on the catalyst surface. The compounds may be adsorbed onto the V/ZSM catalysts and are activated, forming highly reactive complexes that promote mineralization.

The removal mechanisms for nitrobenzene and benzoic acid with V1/ZSM_450_-COP are illustrated in Figure 6C: (1) the compounds are mineralized or degraded to intermediates by direct ozonation. The compounds and intermediates may be partially adsorbed by V1/ZSM_450_. (2) Dissolved ozone adsorbs on the Si-OH-Al active sites and then decomposed to •OHs. (3) Dissolved ozone adsorbs on the V-OHs active sites, where ozone oxidizes V^4+^-OHs to V^5+^-OHs, along with the generation of •OHs. (4) Adsorbed chemicals and intermediates are oxidized or activated via electron-transfer reaction and reduce V^5+^-OHs to V^4+^-OHs. (5) The oxidized or activated species are subsequently desorbed from the catalyst surface and further oxidized by •OHs and molecular ozone on the catalyst surface and in solution.

#### Reaction Pathways of Chemicals in ZSM-5 Zeolites Catalyzed Ozonation

The degradation products of nitrobenzene and benzoic acid after V1/ZSM450-COP treatment at 5, 10, and 15 min were determined using GC-MS. For nitrobenzene, the products were o-nitrophenol, m-nitrophenol, p-nitrophenol, and phenol after 5 min treatment; 4-nitrocatechol and 1, 2, 4-trihydroxybenzene after 10 min treatment and; 1, 3, 4-trihydroxy-6-nitrobenzene, alcohols, aldehydes, and ketones after 15 min treatment. For benzoic acid, the products were phenol after 5 min treatment; 1, 2, 4-trihydroxybenzene after 10 min treatment; alcohols and; aldehydes and ketones after 15 min treatment.

**Figure 7:**
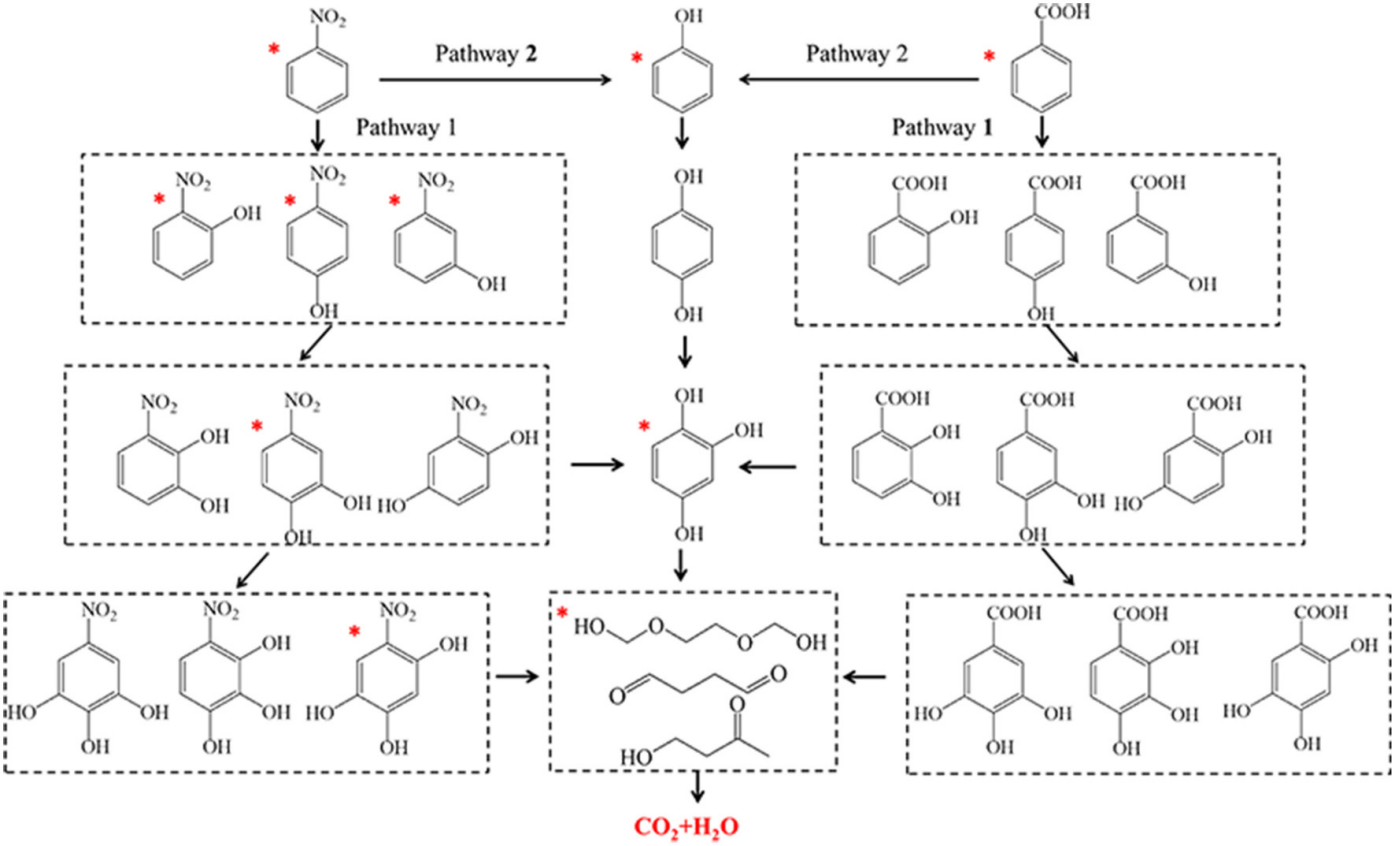
The proposed general reaction pathways of nitrobenzene and benzoic acid in solution. The compounds with an asterisk have been detected.

Two reaction pathways for the mineralization of nitrobenzene and benzoic acid during V1/ZSM_450_-COP are illustrated (Figure 7) based upon the observed intermediate products. For nitrobenzene, the •OHs interacts with nitrobenzene (pathway 1) via an electrophilic addition on a-, b-or g-carbons. Then o-nitrophenol, m-nitrophenol, and p-nitrophenol are produced after 5 min (Di Paola et al., 2003). Nitro (-NO_2_) is an electron-donating functional group, that can increase electron densities at the meta positions, facilitating the •OHs electrophilic addition on α- β-or γ-carbons. The peak intensity of nitrophenols was quantitatively observed as: m-nitrophenol > o-nitrophenol > p-nitrophenol. The •OHs electrophilic addition on nitrophenols then produces 4-nitrocatechol and 1, 2, 4-trihydroxybenzene after 10 min treatment (Zhao et al., 2008). The •OHs electrophilic addition occurs continuously to 4-nitrocatechol, forming 1, 3, 4-trihydroxy-6-nitrobenzene. Then a ring-opening reaction, generating alcohols, aldehydes, or ketones occurs after 15 min treatment. For benzoic acid, the •OHs electrophilic addition (pathway 1) occurs with benzoic acid and forms hydroxyl-benzoic acids, dihydroxy-benzoic acids, trihydroxy-benzoic acid, and tetrahydroxy-benzoic acids (Albarrán and Mendoza, 2018). Nitrobenzene and benzoic acid both follow a similar degradation pathway (pathway 2). Molecular ozone attacks nitrobenzene and benzoic acid via a nucleophilic addition, producing phenol after 5 min (Kasprzyk-Hordern et al., 2003; Nawrocki and Fijołek, 2013). Additional electrophilic additions on the aromatic ring of phenol generate hydroquinone and trihydroxybenzene (Brillas et al., 1998; Goi et al., 2004). The stepwise hydroxylation of nitrobenzene, benzoic acids, and phenol is the dominant reaction process (Albarrán and Mendoza, 2018). The •OHs and ozone then further react with the aromatic ring, forming alcohols, aldehydes, ketones, and carboxylic acids. The final step in this process results in mineralization to CO_2_ and H_2_O. The surface reaction additionally contributes through surface adsorption.

## Conclusion

V oxides modified NaZSM-5 zeolites (V/ZSM) were investigated for use in the COP treatment of nitrobenzene and benzoic acid in solution. V/ZSM-COPs significantly improved TOC removal in comparison to single ozonation, from its catalytic activity. V1/ZSM_450_ (0.7 wt% of V loading and 450°C of calcination) exhibited the highest activity among the developed catalyst compositions. The V1/ZSM_450_-COP increased the mineralization rate of nitrobenzene and benzoic acid by 50 and 41% in comparison to single ozonation. The catalytic activity of V/ZSM catalysts result from a combination of surface multivalent V oxides, Si-OH-Al framework structures and the interactions that occur between them. The co-existing metal V oxides (V^5+^ and V^4+^) are the main active sites that promote •OHs generation via electron transfers between multivalent V oxides and ozone molecules. Indirect oxidation of •OHs, surface reactions and the direct oxidation by ozone are the mechanisms responsible for the TOC removal. This study shows the potential of V/ZSM zeolites in COPs of ROCs in water.

## Data Availability

The raw data supporting the conclusions of this manuscript will be made available by the authors, without undue reservation, to any qualified researcher.

## Author Contributions

QW and CC conceived and designed the experiments. YX performed the experiments. BY, QL, YK, and YT interpreted and analyzed the data. HY and YL contributed reagents materials analysis tools. BY, QL, and CC wrote the manuscript.

### Conflict of Interest Statement

The authors declare that the research was conducted in the absence of any commercial or financial relationships that could be construed as a potential conflict of interest.
